# Remembering, Reflecting, Reframing: Examining Students’ Long-Term Perceptions of an Innovative Model for University Teaching

**DOI:** 10.3389/fpsyg.2020.00565

**Published:** 2020-03-31

**Authors:** Giuseppe Ritella, Rosa Di Maso, Katherine McLay, Susanna Annese, Maria Beatrice Ligorio

**Affiliations:** ^1^Department of Education, Faculty of Educational Sciences, University of Helsinki, Helsinki, Finland; ^2^Department of Humanities, Social Sciences and Cultural Industries, University of Parma, Parma, Italy; ^3^School of Education, The University of Queensland, Brisbane, QLD, Australia; ^4^Department of Educational Science, Psychology, and Communication Science, University of Bari, Bari, Italy

**Keywords:** innovative model, blended learning, collaborative learning, transfer, collaborative and constructive participation model

## Abstract

This article presents a follow-up examination of 10 iterations of a blended course on educational psychology and e-learning carried out at the University of Bari. All iterations of the course considered in this study were designed using the constructive and collaborative participation (CCP) model. Our main research questions are: What are the students’ long lasting memories of this course? How do the students use the skills and the competences acquired through the course across an extended period of time? In line with these research questions, the aims of this investigation can be summarized as follows: (i) to understand the students’ perceptions and long lasting memories of the course and (ii) to investigate the transfer of skills and knowledge across an extended period of time, based on a self-reported survey. The analysis was carried out by administering the survey to all 196 students who took part in the course in the 2005–2015 decade. 96 participants responded to the survey. The survey is designed to collect data in two areas. First, the memories related to the course and second, the way skills and content knowledge acquired during the course have been transferred to and used in other contexts after the course ended. The data were analyzed using a mixed methods approach, which revealed trends in the responses across the decade. In general, participants remembered the teaching methodology and often recalled specific activities such as Role Taking and the creation of products through group-work. These activities and approaches seemed to provide significant learning opportunities for the students. Several students also recalled key concepts and content knowledge acquired during the course. In relation to transfer of skills, participants tended to reuse mostly transversal skills, such as communicative and organizational skills, especially in work contexts. Further, about half of the respondents reused the content knowledge of the course. This analysis is valuable because it allows us to understand the aspects of the model that are significant for the students in the long term, and to discover and interrogate the acquisition and transfer of skills useful for the students’ personal and professional lives beyond the academy.

## Introduction

The advent of digital technology is contributing to profound transformations in many spheres of life, requiring new literacy frameworks, and the development of novel practices of teaching and learning. Nevertheless, educational institutions have often failed to fruitfully transform their practices of teaching and learning (Hakkarainen, 2009) and to align with the evolving needs of the digital society. Some authors argue that this claim is also compelling for higher education, where universities and students have been facing challenges “in making ‘good’ use of digital technologies” (Henderson et al., 2015, p. 2). One example is the case of interactive whiteboards, which in many schools and universities are part of the normal technological toolset available for teachers and students. However, research shows that their usage is often limited to traditional learning practices that could be carried out on normal boards (Gursul and Tozmaz, 2010; Ritella and Sansone, 2020). Therefore, the capacity of educational systems to prepare learners for complex twenty-first century life and work is under increasing scrutiny. The importance of this point is emphasized by research showing significant interconnections between educational achievements, occupational success and well-being (e.g., Samuel et al., 2013). In 2017, The European Commission affirmed the need to strengthen European identity through education and culture, foregrounding the responsibility of educational systems for students’ development of the necessary tools to thrive in the new paradigm of a knowledge society. These tools, which are often defined in terms of transversal competences or career and life skills (CLS), include: flexibility and adaptability; initiative and self-direction; social and cross-cultural skills; productivity and accountability; and leadership and responsibility (Kivunja, 2015). However, whether universities are employing teaching practices that help students to develop the transferable skills and competencies needed in a rapidly evolving society is questionable.

To address this issue, we argue that it is crucial to examine student perspectives. Indeed, students’ interpretations and sensemaking concerning educational activities are crucial both for academic achievement (Schneider and Preckel, 2017) and for the transfer of skills and knowledge (Engle, 2006). For example, research shows that discursively framing a learning task in connection to broader contexts where students use what they learn may support the transfer of learning (Engle, 2006). In particular, the space-time context in which a learning task is situated seems to play a role in mediating the students’ interpretations concerning learning situations (Ritella et al., 2017). Beyond the interpretations of single learning situations, students’ interpretations of the implications of their academic activities for life after graduation need to be considered. A few large-scale studies show that most students often feel that their academic studies have not prepared them well for their professional life. For example, a McGraw-Hill Education (2016) of a large and heterogeneous sample of American university students found that only 21% feel very prepared to start their professional career. In Italy, the situation is similar. The Eighth Eurostudent Survey (2016–2018), focused on Italian university students, provides a problematic assessment of students’ professional knowledge acquisition. While four out of five are satisfied with their theoretical preparation, less than 50% feel professionally prepared. Thus, there is a need for higher education to better fulfill its *trait d’union* function between educational and professional spheres, helping students to develop “the skills most in demand in the 21st century workplace” (Kivunja, 2015, p. 1). This involves redefining the aims of higher education and reevaluating strategies to accomplish these aims. This is a complex and multidimensional task that arguably involves challenging and undoing decades of what many scholars characterize as misguided policies that have, across the globe, cultivated homogeneity, standardization and “testing over teaching” (Zhao, 2015, p. 129) – qualities that are at odds with the CLS necessary to flourish in twenty-first century life and work.

Effective psycho-pedagogical models are widely regarded as key to enhancing learning outcomes for contemporary university students (Schleicher, 2011). This involves “creating environments and feedback mechanisms and systems to allow students’ views, learning experience, and their performance to be taken into account” (European Commission, 2013, p. 28). For example, this might be the case in blended and flipped approaches (Wanner and Palmer, 2015); cooperative learning in small groups (e.g., Smith et al., 2005; Gillies, 2007); and in approaches that actively involve students in teaching and learning processes, encouraging student contributions to coursework planning (see Cook-Sather, 2002; European Association for Quality Assurance in Higher Education [ENQA], 2005, 2015). Kelly et al. (2014) contend that learning and teaching processes should be developed in parallel, looking for intersection points between how to teach and how to learn. Furthermore, tools and technologies deployed in the classroom are important; an effective teacher can cultivate a rich, stimulating and appropriate environment for students by knowing how to choose the right tools and methods (Lucena et al., 2018). The role played by digital tools in collaborative tasks is particularly important as mediating tools can significantly affect the collaborative sensemaking of the groups (Ritella and Ligorio, 2016) and, through participation, the identity construction of learners (Annese et al., 2010). However, while it has long been recognized that the new paradigm of twenty-first century life and work demands approaches that provide learners with more autonomy (Steeples et al., 1994) such as those described here, educational innovation in university settings is not easy to implemente.

Tadesse and Gillies (2015) argue that innovating to achieve “instructional conditions that promote quality learning [is] challenging for many higher education teachers” (p. 1) for whom traditional lecturing remains the most common approach. Some research indicates that innovation is only possible when both political and corporate stakeholders are involved and aligned (Etzkowitz, 2008; Mowery et al., 2015). Others contend that university teaching can be improved by focusing on training skills and supporting teaching with purposely trained tutors (Muukkonen et al., 2005; Sansone et al., 2016). Through a systematic review of 38 meta-analyses investigating 105 correlates of achievement, Schneider and Preckel (2017) discussed the variables that are associated with achievement in higher education. In their account, the mere presence of technology has little effect compared to other variables such as social interaction, meaningful learning, and assessment. This reflects research showing that digital tools do not automatically improve educational practices, nor affect learning by themselves (Säljö, 2016). The effects of technology on education also depend on how tools are integrated into practice. In particular, assessment practices are crucial for any significant shifts in university teaching structure. Indeed, when educational practices change, a robust evaluation system is needed, able to account for different dimensions, including the various elements of a course (Gatignon et al., 2002). However, the typical elements that are usually considered when discussing teaching methods are not able to ensure high quality teaching practices. Indeed, one of the most interesting findings of the review conducted by Schneider and Preckel (2017) is that strong moderator effects were found for all the teaching methods considered. This indicates that how a method is implemented impacts on achievement. For example, “[t]eachers with high-achieving students invest time and effort in designing the microstructure of their courses, establish clear learning goals, and employ feedback practices” (Schneider and Preckel, 2017, p. 565). Going beyond examining general features of the teaching methods and technological aspects is therefore valuable because interrogating the details of course design (e.g., orchestration of activities, tools, and strategies) can support deeper understanding and more successful implementation of future iterations.

In response to these challenges, this paper first describes in detail the organization of a course based on the CCP model. The perceptions and memories of students, especially concerning the capacity of the course to foster the transfer of CLS across multiple professional and personal contexts, is then explored. In particular, the analytical focus is on the long-lasting memories and long term effects of the course concerning the transfer of competences and skills. In other words, our goal is to discuss if and how the course under scrutiny has had any significant impact on the students’ lives in the long term, and how the students remember it several years after completion. We argue that current literature on the topic tends to focus on the short term and is mainly based on data collection carried out immediately after the completion of a learning experience. To our knowledge, there are not existing surveys specifically designed to examine these issues on timescales beyond a few months. For example, research on students evaluation of teaching (SET) is usually based on surveys carried out immediately after the end of the course. This approach is not well suited to examining the long lasting memories that the students can retrieve several years after completion of a course, nor the long term transfer of competences. Similarly, the survey developed by Maul et al. (2017), which allows examination of how students perceive their school experience as “connected” with their interests and with their life out of school, is meant to be administered when the students are participating in the learning programs investigated. In addition, although this survey addresses issues relevant to our study, it is not designed for higher education. Our review of the existing literature led us to develop a survey specifically designed for our study (Supplementary Appendix 1).

In order to address this research gap identified in the literature, our analysis examines the elements of the course that the students remember as most significant several years after the completion of the course and investigates the role of the learning environment in developing and supporting the re-use of both soft and professional skills beyond graduation. The elements retained by the students, as well as the self-reported transfer of skills to other contexts in the longer term, can be considered crucial dimensions to evaluate course design based on the CCP model.

## The Model of Constructive and Collaborative Participation

The students under scrutiny in this research have participated in a university course titled “Psychology of education and e-learning,” offered at the University of Bari, in Italy. The course takes a blended approach using the collaborative and constructive participation (CCP) model, developed over more than 10 years of consecutive application to several higher education courses (see Ligorio and Sansone, 2009; Ligorio and Annese, 2010; Ligorio and Cucchiara, 2011). The CCP model conceives learning as the co-construction of knowledge and aims to support students to develop new ideas through the creation of both individual and group products (Cucchiara et al., 2014). Drawing on social constructivism (Kelly, 1955; Berger and Luckman, 1966; Shotter, 1993; Potter, 1996; Gergen, 1999, 2001; Scardamalia and Bereiter, 2006), this course requires students to build knowledge through actively producing meaning, products and forms of interaction, negotiation, and social collaboration. Thinking is not considered as a private or individual process; rather, it is distributed or “stretched across” people and the environment, artifacts, and technological tools mediate the relationship between individuals and the learning context (Suchman, 1987; Lave, 1988; Hutchins, 2001; Ritella and Hakkarainen, 2012). Learning is a complex system in which the relationship between the subject and the object is mediated by artifacts (Engeström, 1987) and by social factors such as teamwork and collaboration (Dillenbourg, 1999) that occur both online and offline (Graham, 2006). The social dimension is crucial to this process; people learn through interaction with other members of a community (Brown and Campione, 1990; Wenger, 1998). Grounded in these theoretical underpinnings, the course is comprised of alternating online and offline activities, which are distributed across five or six modules, lasting an average of about 10 days each. Each module begins with face-to-face lectures, during which the teacher introduces the content of the module, and ends with the students jointly negotiating a research question that guides all subsequent module activities. The modular structure allows for easy comparison across cohorts, with each module operating as a milestone within the course. In the design, implementation and evaluation of the course, the model considers the intervention of purposely trained professional tutors, who act as mediators between the teacher and students. The course activities can be summarized as follows.

### Independent Individual Activities

These are activities that students can perform alone, without the support of other peers. Individual activities support self-evaluation strategies, including metacognitive reflection upon what has been done and what to do next. Two activities are included in this category:

• An individual e-portfolio which includes personal information and material such as photos, reflections, links to Facebook pages and blogs. At the end of each module, students include this material in their e-portfolio, record what they feel they have learned, and outline their goals for the next module. These two latter aspects of the e-portfolio are based on the concepts of the actual zone of development and the proximal zone of development conceived by Vygotsky (1986). This tool is discussed in more detail in a later section.
• Compilation of a self-assessment grid. At the end of each module, students identify the specific and transversal skills acquired. The grid is comprised of questions aimed at developing critical self-assessment and recognition of the skills learned.

### Individual Interdependent Activities

These are activities performed individually within a group. They are designed to support individual responsibility within a social context and provide a structure for student social participation and include:

• Writing a review. Groups of students called “expert groups” are formed. Within these groups, to each student is assigned a specific learning material (e.g., a book chapter, a scientific article, a website, etc.). Students can discuss the material but are ultimately individually responsible for writing a critical review (e.g., summary of key information, strengths and weaknesses, etc.) to inform the subsequent group discussion. Each review is an individual but interdependent activity because, once the reviews are complete, the expert groups are dissolved and new groups are formed. Students from the different expert groups now form a new group where they use the reviews to build a shared answer to a previously negotiated research question. This activity is inspired by the Jigsaw model (Aronson et al., 1978; Aronson and Patnoe, 1997) and it is appropriately adapted to the blended nature of this course by including web-forum discussions and online group work.
• Role Taking. Inspired by the work of Strijbos and Weinberger (2010), Role Taking requires each group member to take on a specific tasks and responsibilities (Hare, 1994), aimed at supporting individuals to achieve a shared objective (Topping, 2005). Student participation is clearly structured to (i) improve individual satisfaction (Zigurs and Kozar, 1994); (ii) empower students with a sense of individual responsibility; (iii) support group cohesion; (iv) stimulate awareness of the interational processes (Mudrack and Farrell, 1995); and (v) support group dynamics. Examples of roles implemented within the course and assigned in turn to students are:

• E-tutor: The e-tutor coordinates the group discussion, manages times and spaces (when and where the group will meet), and monitors the development of the other roles. The student who performs this role must have a clear understanding of the objectives of any group discussion and of the related tasks. The e-tutor becomes the temporary leader of the group and must deploy suitable communication strategies to stimulate collaboration. This role is designed to (i) keep discussion focused on common objectives; (ii) monitor deadlines; (iii) be aware of the functions and affordances of the virtual space; (iv) regulate possible conflicts between group members; (v) manage unexpected events; and (vi) balance focus on the task with attention to relationships.
• Synthesizer (S): The synthesizer’s role is to summarize the group discussion. This metacognitive role requires the student to analyze and describe the group dynamics and methods of discussion rather than engaging with subject matter. In particular, the synthesizer considers how discussion progresses, from facts and data to ideas and knowledge building. Students who take on this role develop the skills to carefully and critically review discussion, as well as the capacity to identify and manage the dimensions that can help or hinder the group’s progress.
• Product Manager: The student in this role manages and monitors the process of building the collaborative products (further discussed in the following section). Taking this role requires the student to develop their capacity to coordinate and supervise group work to successfully develop a collaboratively designed and built product.

Students rotate through these roles from one module to the next so that all students experience as many different roles as possible. Through Role-Taking, ways of participating that would not otherwise be experienced are encouraged. Students are assigned to the roles randomly.

### Small Group Activities

These activities are organized so that success can only be achieved if students work collaboratively in their small groups. There are two types of activities in this category:

• Jigsaw group activities. Students are first required to read all the critical reviews produced by the participants and then to discuss them, searching for connections to their research question. Discussion usually occurs online via web-forum but can also be interspersed with face-to-face discussion, if the group prefers. The progressive inquiry model (PIM) (Hakkarainen and Sintonen, 2002) guides both expert and Jigsaw group discussions. Within this model, learning is conceived as a process of investigation, which begins with a large and general question – in our case, the research question underpinning the module. From here, the focus shifts to critical assessment of the various dimensions of the issue under discussion. To this end, students are encouraged to search for further material that will develop and deepen the problems that have emerged. Critical thinking is encouraged by comparing different ideas and divergent material. The aim is to distribute cognition among all participants.
• At the end of the discussions within the Jigsaw groups, students are required to collaboratively create a joint product. This can be a text summary, a concept map or any product that the group considers suitable for the content.

### Plenary Activities

Plenary activities aim to involve all course participants by interconnecting the groups to produce a collective product. All groups are required to build an object that synthesizes what that group has learned. To do this, we often used a grid of indicators that made visible the salient features of e-learning courses. First, all the groups jointly singled out the crucial dimensions of e-learning (i.e., modalities of collaboration, features of the platforms, etc.). After that, each group takes up one dimension and identifies aspects of the course in which that dimension is evident. Finally, all the indicators are compared and organized into a grid that guides observations of e-learning courses in ways that support understanding of its features.

Other approaches to a final product could include a text, a concept map, or a multimedia product summarizing the content of the course. In any case, the plenary work should allow the transfer of knowledge and skills acquired within the smaller groups to larger ones, creating dynamics of collaboration, and meta-reflection on activities, as well as creating space for students to grapple with diverse perspectives.

## Ongoing Assessment of the Course

As discussed above, assessment is a core feature of innovative educational practice, which has a strong impact on students’ achievement. Therefore, the CCP model involved a careful design of the assessment process. A substantial proportion of research into assessment calls for students to be active participants so they can understand what and how they learn, and to test the efficacy of teaching. To achieve both these aspects, two types of assessment have been considered in the design of the course: assessment for learning (AfL) – also known as formative assessment, assessment as learning, and learning-oriented assessment (Carless, 2016) – and self-evaluation. AfL can be distinguished from assessment of learning or summative assessment, which focuses on grading or marking student work. Promoting student learning rather than grading is the first priority of AfL; this is achieved by helping students reflect on what they know and what they can do, then use this understanding to identify gaps in their knowledge, connect concepts, and face new problems (van Dinther et al., 2015). AfL is a multi-dimensional vision of assessment that conceives cognitive, emotional, affective and social aspects as integral elements of the learning process, and seeks to make visible students’ thinking and reasoning skills using a variety of tools (Astleitner, 2018). Two of the most widely used tools for formative assessment are e-portfolios and case-based assessment.

The second type of assessment, self-evaluation, has been the object of some scholarly disagreement. According to Topping (2003), self-evaluation should be considered a formative assessment technique, while others conceive self-evaluation as a tool for encouraging students to take responsibility for their own learning (Brown and Harris, 2014). Either way, self-evaluation requires students to assess aspects of the learning process (for example, as members of a group) they are involved in and of the products they build (for example, the task they are working on). Zimmerman (2001) considers self-evaluation a key component of the broader ability to self-regulate. Self-evaluation is closely connected to the capacity to manage learning processes, as well as to meta-cognitive and motivational aspects through which personal skills are deployed to control learning outcomes. Our contribution takes into account both approaches. In the following section, we describe and analyze data generated through longitudinal follow-up study “cohorts” – groups enrolled in the same course but in different academic years.

Given these premises and definitions, the CCP model approaches assessment to sustain students’ reflection upon their own learning journey and on how they learn most effectively. We already reported how during the course, self-assessment and peer-assessment (Topping, 2005) are facilitated through specific tools and interactive moments. The goal is to equip students with critical skills in terms of their learning methods, participation in group-work and, ultimately, their results. The main goal is to cultivate self-monitoring – a critical twenty-first century competency that both empowers and supports life-long learning. The tools used are:

• The e-portfolio. As described earlier, at the end of each module students are asked to review what they learnt and set personal goals for the upcoming modules. At the end of the course, the e-portfolio provides students a concrete artifact of their learning journey and helps them identify knowledge, skills, and attributes for inclusion in their curriculum vitae or professional social networks profiles, such as LinkedIn. The e-portfolio is deliberately structured to promote self-regulation and self-assessment. Cultivating student awareness of their current and proximal zones of development promotes autonomy and a sense of agency (Bruner, 1996). This helps students focusing less on performative aspects, like summative assessment, and more on evaluating the strategies they have deployed to achieve their goals. Further, explaining the criteria they used to select skills and competences for inclusion in the e-portfolio supports self-assessment (Brown and Harris, 2014).
• A self-assessment record. At the end of each module, students, teacher and tutors record all course activities (individual reviews, collaborative products, role taking, and discussions, etc.). Students must score each activity on a scale from 1 to 5 in relation to (i) how much they believe the activity supported their learning of content and skills, and (ii) the factors they believe contributed to the success of the activities. The aim is to stimulate students’ meta-cognitive processes of critical reflection on their own abilities and performance. The teacher and the tutors also fill in the same sections, so students can compare their perspectives with their own self-assessment. By looking at the score obtained across the modules, students can trace their evolution throughout the course.

At the end of each module, students are invited to read the assessment record and comment on it in a dedicated forum. This forum is an opportunity to “wonder,” reflect, and appreciate achievements as well as to express doubts and ask questions in an extended discussion between peers, professional tutors and the teacher. This type of discussion aims to help students improve self-assessment, adjust their learning and participation strategies, taking them closer to self-regulation.

## Research Questions

Our main research questions are:

1. What are the students’ long lasting memories related to this course?
2. How do the students use the skills and the competences acquired through the course across an extended period of time?

In line with these research questions, the aims of this investigation can be summarized as follows: (i) to understand student perceptions and long lasting memories related to the course; and (ii) to investigate the transfer of professional skills and knowledge across an extended period of time, based on a self-report survey. We were also interested in whether the students’ perceptions vary over time. To this end, all the students who took the course in the decade 2005–2015 were considered. During this decade, the structure of the course remained almost unchanged, making these iterations of the course comparable. The decision to include several iterations of the course across a decade also supported answering our research questions. Interrogating students’ long lasting memories and the transfer of learning over an extended period of time justifies the inclusion of students who had completed the course three to 13 years before data collection. We considered the iterations of the course held at least 3 years before because we expected that this span of time would allow the consolidation of long term memories and it would also allow the students to experience several educational or professional contexts in which to use the skills, knowledge and competences acquired during the course. In addition, the longitudinal dimension helps us to examine the long term effects of some actions related to group management (discussed in the section “Domain 2: Skills” of this article) that were implemented during the course to improve group dynamics.

## Participants and Data Collection

Data collection was carried out through an online questionnaire using Google forms (see Supplementary Appendix 1). The questionnaire link was sent to all 196 students who had completed the course. 96 students (49% of the sample) answered the questionnaire. Of these, 81 were female and 15 were male. This composition reflects the gender distribution within the course, which is part of a Master of Psychology that, in the Italian context, attracts more females than males. The unbalanced gender distribution did not represent a concern for the design of our study because we did not plan to examine gender differences. The average age was 28 years. The number of participants over the 10-year period has steadily increased each academic year, as shown in Table 1.

**TABLE 1:** Number of participants and respondents per academic year.

Academic year	Number of participants	Number of respondents
2005/2006	10	2
2006/2007	10	3
2007/2008	16	6
2008/2009	14	9
2009/2010	25	10
2010/2011	19	8
2011/2012	16	7
2012/2013	17	7
2013/2014	39	30
2014/2015	30	14

The questionnaire was constructed in stages. First, a draft was prepared by the lecturer in consultation with two experts in designing questionnaires. The draft version was administered to 10 students randomly chosen from those enrolled in the course in the last three academic years. Immediately after completing the questionnaire, the teacher and experts met one-on-one with these students to seek their feedback. Specifically, participant students were asked to explain how they interpreted each item and whether they could suggest improvements to the questionnaire. After collecting this feedback, a second version of the questionnaire was prepared in consultation with the two experts. The second version was also shared with the course tutors. This resulted in further comments and suggestions that led to the final version of the questionnaire.

The final version of the survey consisted of two domains: The first focused on memories of the course, and the second focused on the reuse of the skills and knowledge acquired during the course in other contexts. To investigate memories, an open question was used to encourage free expression. In order to investigate to what degree skills and knowledge had been reused, we constructed 27 items to which participants responded on a three-point Likert scale (1 = little, 2 = enough, and 3 = a lot). Finally, to investigate the contexts in which skills and knowledge were reused, we deployed two multiple-choice questions asking participants to indicate the online and the offline contexts where they had mostly re-used the skills and knowledge acquired through the course.

## Data Analysis

The questionnaire responses were first analyzed considering the whole corpus of data; that is, all the academic years involved. This provided a broad overview of the students’ perceptions. Subsequently, the questionnaires were grouped into the following three clusters: (i) academic years 2005–2006, 2006–2007, and 2007–2008; (ii) academic years 2008–2009, 2009–2010, and 2010–2011; (iii) academic years 2011–2012, 2012–2013, 2013–2014, and 2014–2015.

A mixed methods approach was deployed. Multiple-choice and Likert scale items were quantitatively analyzed by calculating frequencies and percentages. For this purpose, the 27 items about skills and content have been grouped into the six following categories:

• Organizational skills (how to work toward a common goal; how to manage work deadlines).
• Communication skills (how to communicate effectively during collaborative work; how to participate in the creation of collaborative products).
• Managing group dynamics (how to negotiate between different points of view; how to observe group dynamics).
• Academic skills (how to write academic texts; how to find useful and reliable material online).
• Content about e-learning (how to assess online courses; how to operationalize theoretical constructs related to e-learning).
• Self-assessment skills (how to enhance their own skills; how to assess their own learning content and strategies).

The open question on memories was analyzed by building a system of categories using the Grounded Theory methodology (Glaser and Strauss, 1967). First, all responses were analyzed by two researchers who worked independently to code the data without predetermined categories. After independent analysis, the coders compared the categories that they identified and 90% agreement was reached. The remaining categories were discussed with a third researcher until 100% agreement was reached. Each memory has been segmented by isolating specific units of content and the coding was applied to each segment. In this way, more than one category could be assigned to each answer.

The categories identified are described in Table 2, including examples from the data corpus for each category. The three independent researchers reached unanimous agreement to confirm each code assignment. Once the system of categories was defined, an analysis of the frequencies and percentages on the whole sample was carried out. Later, the three clusters previously formed were compared to understand differences between them.

**TABLE 2:** The category system for the analysis of memories about the course.

Category	Description	Examples
Teaching methods	References to the educational models used i.e., activities, group work, individual work, Role Taking, objects produced	*I remember a particularly interactive and dynamic course, where the groups were the privileged places of exchange, comparison and construction of new knowledge through the enhancement of the various roles covered by each member (as tutor, map manager), alternating moments of work online to moments for face-to-face discussion and collaboration*
Technological devices	References to the tools and technologies used during the course	*I remember we used any type of online tool; chat, virtual whiteboards, videoconferences, forums, ebooks*
Skills	References to soft and professional skills acquired during the course	*In addition, critical thinking, the ability to synthesize, to collaborate and to construct knowledge have become my skills*
Group dynamics	Memories related to processes and group dynamics	*Group dynamics, cooperation, collaborative learning, respect for group norms, exchange and relationship*
Educational content	Memories of syllabus content	*I remember the definition of e-learning and how to go from theory to practical implications*
Generic comments	Generic memories of the course	*Excellent course. Innovative, motivating and creative*

## Results

The analysis of students’ memories is useful to identify key aspects of the course that participants recall and the meanings that the students associate to them. The examination of responses concerning skills and knowledge, and the contexts of their reuse, helps us identify participants’ perspectives about which skills were transferred to other contexts and where they were re-used. Together, these two aspects provide rich insight into the student experience of this course, as well as into the perceived effectiveness of the course design in fostering the transfer of knowledge and skills. Further, these data can also be used to improve the next iterations of the course. In the following sections, we first present results concerning participants’ memories of the course. In the subsequent section, we report the data in relation to learning, skills and re-use. In both sections, we consider the totality of the sample in the first instance, followed by an analysis of the three clusters. For an overview of the data see Supplementary Appendix 2.

### Domain 1: Participants’ Memories of the Course

**FIGURE 1:**
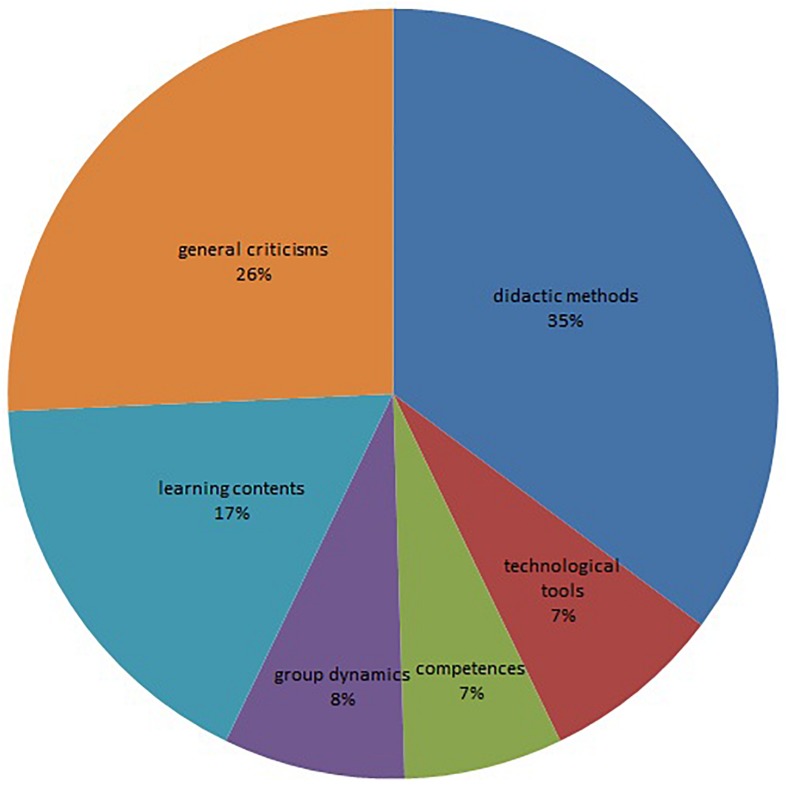
Distribution among the whole sample. Categories of course memories.

The analysis of the participants’ memories of the course shows that, beyond a series of generic comments on the course (26%), the participants mostly remember the teaching methodology (35%), and the educational content (17%). The technological tools used (7%), the skills acquired (7%) and the group dynamics (8%) (see Figure 1) reached lower percentages and are more or less at the same level. Within the category “Teaching Methodology,” many have qualified the method as innovative and exciting, as we can see from the following excerpts:

Excerpt n. 1: “*The learning modality: absolutely innovative, interactive, stimulating. The content were learned using them in the online platform context*.”
Excerpt n. 2: “*I remember the innovative way of conducting the course and the exam, the collaborative atmosphere, the blended approach, the scaffolding of the tutors and the challenging objectives of the course.”*

Considering that this was the first experience of blended courses during their academic studies, this is not surprising. Nevertheless, the sense of innovation that has been perceived by the students might have contributed to their learning. Indeed, it is known that novelty can have multiple effects on cognition, including the enhancement of learning (Schomaker and Meeter, 2015). A close analysis of this category indicates that the activities of Role Taking (RT) (27%) and the creation of products through group work (22%) were particularly memorable to participants. With regard to RT, this approach appears to have elicited emotional and cognitive involvement that stimulated students to activate new forms of reasoning and interaction, as we can see in the following extracts:

Excerpt n. 3: “*I perfectly remember the importance of the roles that, in my opinion, have made this course closer to us students making us feeling really part of a community. Having covered several roles has allowed me to experience the different dynamics that can emerge within an online group and to understand both the negative and the positive aspects.”*
Excerpt n. 4: “*I remember that there was the role of the critical friend, whom I liked very much and it had greatly stimulated my reflection.”*

The role of the Critical Friend, mentioned in excerpt n. 4, has the task of offering constructive criticism on another student’s products. This role is designed to promote critical thinking and argumentation, as well as relational skills and the capability to empathize with the author of the product. We suggest that the design of tasks based on the RT approach might have been one of the elements characterizing the course design for the students and contributing to the students’ learning achievement. In particular, the memories of the students might signal that RT supported the development of a range of inter and intrapersonal skills that are still considered relevant and useful for them at the moment of the data collection, which took place several years after the end of the course.

Concerning the collaborative activities, the participants most often cited the construction of the conceptual maps. Participants saw this activity as valuable because it promoted skills related to effective summarizing and synthesis:

Excerpt n. 5. “*The construction of maps, the importance of syntheses and discussions with others.”*
Excerpt n. 6. “*I remember we were working on collaborative conceptual maps in every module, which for me was a very important activity for summing up the content of the module.”*

**FIGURE 2:**
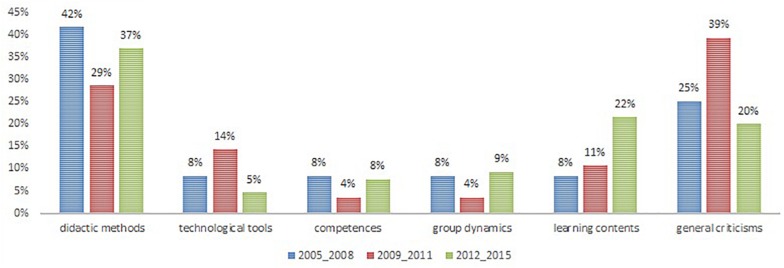
Distribution comparing clusters. Categories of memories across the clusters.

When we compared the results across the three different clusters, we detected a similar trend in all clusters although there were slight differences, as shown in Figure 2. From this figure, it is clear that the course content was best recalled by the 2012–2015 cluster, probably because this is the most recent one. Nevertheless, the memories reported by the students from the clusters 2005–2008 and 2009–2011 show that at least some of them have a clear memory of some of the concepts that were addressed during the course, such as virtual communities, knowledge building, interactivity, blended learning, etc. Below we report two excerpts that were categorized with this code:

Excerpt n. 7. “*I remember the concept of collaborative learning and knowledge building.”*
Excerpt n. 8. “*Virtual communities of learning, interactivity, learning by doing, multimedia, blended learning, virtual classrooms, semantics web.”*

### Domain 2: Skills

**FIGURE 3:**
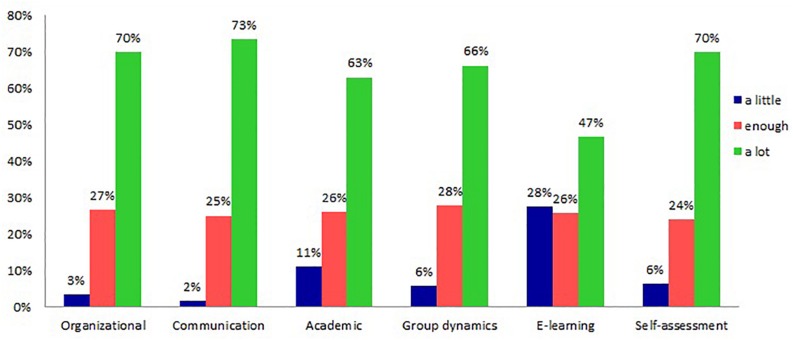
Distribution of categories with higher score. Reused skills and knowledge acquired during the course.

The analysis of skills and knowledge acquired during the course, reported in Figure 3, shows that on average 70% or more of the students re-used communicative, organizational and self-assessment skills, while the transfer of academic skills and skills related to collaboration and group dynamics is reported as high by approximately 66% of the students. Instead, the 47% of the students declare to have reused knowledge and content concerning e-learning. The answers in this domain suggest that the course provided to a high percentage of occasions for developing transversal skills that they have found useful in their subsequent career after graduation, while the specific content of the course has been reused by about half of the students. This might also be related to the different career paths started by the students, since some of them might have chosen careers in other fields of psychology where e-learning is not necessarily relevant.

The skills with the highest percentages of responses relate to effective group communication (73%) and to the ability to participate effectively to create collaborative products (72%). A close examination of these categories reveals that most participants have re-used the ability to be flexible during problematic situations that arise in the group (73%), which is a subset of communication skills considered in the survey. The organizational skills that are reused most frequently are related to the organization of group work toward a common goal (76%). Finally, the self-assessment skill with high frequency of transfer concerns the ability to know how to exploit and maximize one’s own skills (69%).

**FIGURE 4:**
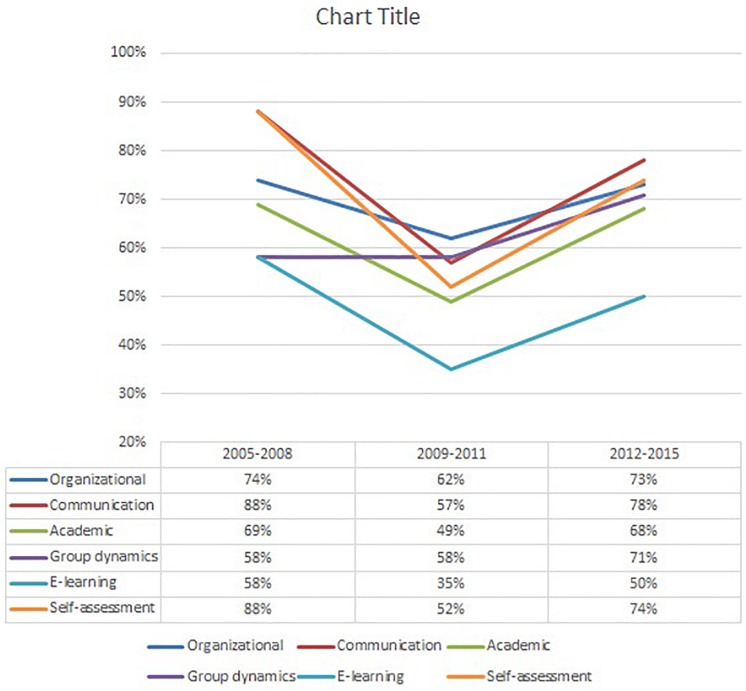
Distrubution of the higher score across clusters. Skills reused divided by clusters.

Figure 4 represents the percentage of respondents who answered “a lot” for each type of competences across the three clusters (2005–2008, 2009–2011, and 2012–2015). As shown in this figure, the participants in the first two clusters reported Organizational, Communicative and Self-assessment skills as the most often reused; this is in line with what emerged from the analysis of the whole corpus of data. The second cluster seems to show a drop in all dimensions apart from the skills related to Management of group dynamics, which in the third cluster increased to 71%. One possible interpretation of this result is that throughout the 10 iterations considered in this study, the teacher carried out specific actions to improve group management, based on previous experience (Annese and Traetta, 2012). Indeed, the management of group dynamics was not easy in the first iterations of the course and the teacher and tutors dedicated extra effort and time to understand how to improve this aspect in future courses. This is well reflected in the data.

**FIGURE 5:**
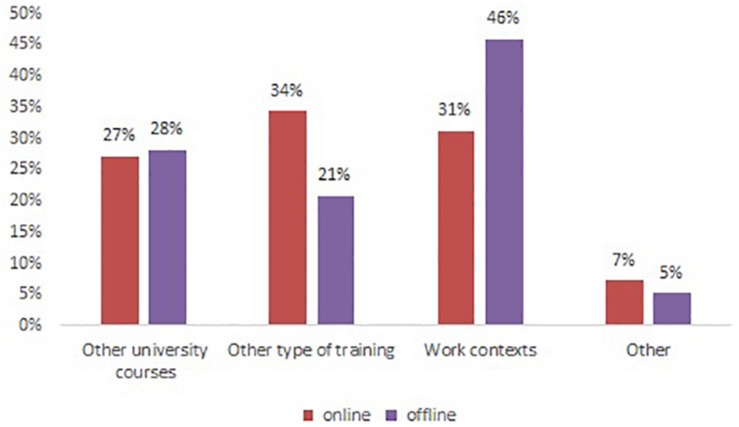
Skills reuse comparing online and offline contexts. Contexts of use of skills.

Looking at the contexts where the students reported to have reused these skills, almost half the participants (46%) reused them within offline work contexts (Figure 5). This finding seems to suggest that this course allows a learning experience highly relevant for work contexts. Instead, only 34% of the students reused them in other contexts of online training.

**FIGURE 6:**
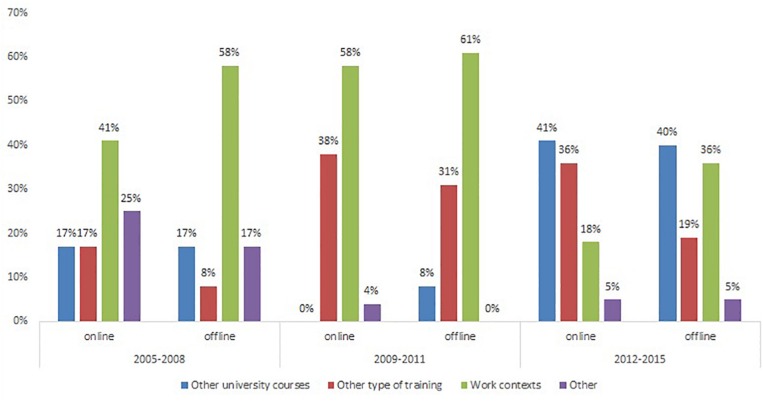
Skills reuse comparing online and offline contexts across clusters. Contexts of re-use divided by cluster.

Comparing the data across the three clusters (see Figure 6), it became evident that for the first and second cluster the acquired skills were mostly reused in work contexts, while for the last cluster the most relevant contexts for re-use were other university courses. This might be interpreted considering that the students belonging to the first two clusters have completed their academic studies earlier than the others and probably had more occasions to reuse their skills at work. The students belonging to the last cluster instead might be still enrolled in a university degree or searching for their first job. Indeed, in the local context of South Italy – characterized by high unemployment rate among young people, even those with a master’s degree – the transition from higher education to the working career might take several years. Therefore, the differences among the clusters seem to capture different dimensions: the first two clusters allow the examination of the transfer of learning from academia to professional contexts, while the last cluster seems to generate more insights about the transfer across multiple learning contexts experienced by the students. Unfortunately, we did not ask to the students any question about their working career, thus this hypothesis needs to be confirmed by future research. Moreover, for the first and third cluster, the reuse of competences takes place more offline (58 and 36%, respectively), than to online courses (41 and 18%). For the second cluster, online and offline working contexts show similar percentages of reuse.

## Discussion

This paper reports our analysis of students *post hoc* perceptions and memories related to a blended university course based on the constructive and collaborative participation (CCP) model. The goal of this research was to understand the students’ perceptions of the transfer of skills learned through the course across an extended period of time, as well as to identify the elements of the course considered significant by the students and thus retained across time. To do this, we sought feedback from former students who had undertaken the course over a 10-year period. The results were generated through a mixed methods approach, in line with other research studies that deploy a mixed methodology for research into blended learning communities (Annese and Traetta, 2011, 2018). The CCP model is based on principles of socio-constructivism and applies a knowledge building approach to teaching and learning. The CCP model also aims to support students to acquire CLS through productive use of several technological tools. These skills include not only academic abilities but also non-cognitive and personal skills. In this course, academic skills such as analyzing and constructing texts are supported through the task of producing critical reviews. However, non-cognitive skills, such as flexibility and adaptability, initiative and self-direction, social and cross-cultural skills, productivity and accountability, and leadership and responsibility (Kivunja, 2015) are equally important. The course aims to support the development of these skills by requiring students to work effectively and productively in both expert and Jigsaw groups and to produce collaboratively designed products, such as concept maps. Additionally, meta-reflection is cultivated through self-assessment opportunities as well as the e-portfolio, approaches that support assessment *for* and not only *of* learning. All these experiences are embedded in the context of belonging to a community, which simultaneously encourages and values each individual’s contribution by exploiting Role Taking theory that, as we experienced, facilitates social inclusion. The results of this study are promising, as a high percentage of participants have reported to reuse these skills in other contexts in the long term. In particular, the fact that 46% of the students transferred some skills to offline work contexts and 31% re-used them in online work contexts shows that they perceive the design of the course as relevant for their professional career. This finding is at odds with the large-scale studies showing that most students feel that their academic studies have not prepared them well for their professional life.

In particular, the analysis of the area of skills in this study illustrates how the application of the CCP model allowed students to experiment with and enact different types of skills in learning situations that in some ways resonates with the work contexts experienced afterward. This alignment between the learning context and the professional situations could have fostered the reuse of transversal skills after graduation, when the students entered professional life. Analysis of these data indicate that the communicative, organizational, and self-assessment skills have been most often reused in professional contexts.

Our results reflect other research into student satisfaction with e-learning courses (Eom et al., 2006) indicating that students highly value interactions between both students and teacher and among peers through self-assessment and teacher feedback. The CCP model supports these interactions by means of several interconnected activities and tools. For example, discussions aimed at the construction of a collaborative product, and meta-reflection on the roles performed and the e-portfolio. Through these varied interactions, students appear to have developed some transversal skills that are relevant and valuable in diverse contexts – educational, professional, and personal. Also Lai et al. (2016) have found that online interaction (between teacher and students and among students) improves the effectiveness of the course in terms of consolidation of knowledge, causing students to “rethink” what and how they have learned.

Considering the significant reuse of organizational skills, the ability to work in a group to achieve a joint goal, and the ability to be flexible during group work, we suggest that the design of the course based on the CCP model has the potential to powerfully cultivate productive attitudes to collaborative work. By focusing on relational competences and group dynamics the CCP model seeks to enhance students’ ability to create and maintain positive social relationships, and in turn to promote students’ psycho-social wellbeing. In our approach, this is supported also by the opportunities for critical reflection and self-assessment; for instance through the e-portfolio, the self-assessment record, the grid of e-learning characteristics, and by supporting and scaffolding individual participation in collaborative groups through Role Taking. In this way, students learn to be flexible and adaptable by drawing on communication skills in their peer interactions, to demonstrate initiative and self-direction, to be productive and accountable for their work, and to be meta-cognitive about their own contributions and approaches to learning. This often leads students to radically reconsider what they know and what they can do, as well as re-evaluating their goals and personal potentiality.

The sequencing and progression of modules, together with the repetition of activities (but in relation to different content), might have helped students consolidate their learning, and envision a personal trajectory. Students gradually became more self-regulated and their capacity to self-assess improved. Certainly, these claims are not new. For example, Eom et al. (2006) found that self-assessment allows students to become “responsible” for their own learning, for instance. Our research adds strength to the contention that the transversal skills learned in the academic context can be retained and transferred to other contexts. Furthermore, by recognizing the relevance of social interaction based on group work, a qualitative change is induced. Students become more capable to face life experiences in general and their well-being is reinforced based on the interlacement between learning and social processes.

In general, the structure of this course has supported not only the acquisition of skills and their reuse, but also allowed the recall of vivid memories of the teaching methodology and the learning activities. Based on the results presented here, we suggest it is reasonable to claim that teaching strategies, such as Role Taking and the creation of group products, can stimulate the student to go beyond the simple acquisition of knowledge by requiring and supporting them to be active participants in learning. Taking on specific roles encourages both cognitive and intra and inter-personal development, while the collaborative creation of group products makes learning a concrete experience and, in our case, seems to support transition to professional contexts. The perception of innovativeness might play a role in this process.

## Conclusion

The CCP model presented in this paper holds promise as an example of innovative teaching for higher education that effectively combines a range of approaches to teaching and learning, sharing a collaborative and constructive vision of learning aimed at supporting students’ development of skills and competences relevant for the students’ lives after graduation. Furthermore, by contributing to significant learning for the workplace, the implementation of course design based on the CCP model can enhance students’ sense of efficacy when facing novel working situations and thus contributes to their wellbeing in the long term. Indeed, as claimed by Salanova (2004), efficacy beliefs are associated with increased psycho-social wellbeing and performance.

Of course, this research is not without limitations. The number of participants in some clusters was lower than others, and there was a higher percentage of female than male participants in all the cohorts, restricting the opportunity to run more advanced statistical tests and to compare gendered experiences. Nevertheless, we argue that the descriptive quantitative analysis combined with the qualitative analysis of the answers to the open-ended questions, allowed us to explore this overlooked topic of investigation and glean valuable insights concerning students’ perspectives over a long-term timeframe.

In relation to the evaluation process of higher education courses, having a proper evaluation tool is essential (Kirkpatrick, 1998; Owen and Rogers, 1999). In the Italian higher education context, SET questionnaires are the only tool available and they are administered right at the end of the courses or at most, a few weeks later. In those questionnaires, students cannot comment on their learning processes and it is impossible to assess the learning impact of the courses across extended periods of time. Therefore, additional tools are needed to examine the multifaceted experience of students over longer timeframes; these are often overlooked in current research. The CCP model, including teaching strategies such as Role Taking and group work, is expected to trigger specific learning processes having long-term effects in terms of CLS development. Therefore, there is a need to develop strategies to examine such long-term effects. The mixed methodological approach used in this paper allows for assessment of the “historical effect” (Green, 1977) of the course; that is, the long term impact of the learning process.

Although our study does not allow for generalizability of findings due to the limitations discussed above, it does support discussion of the long term effects of the learning model for the participating students. Further, this research provides feedback to different stakeholders of the educational context: teachers, learners, and policy makers.

First, this type of research allows teachers to reflect on their professional decisions, to identify strong and critical aspects of the psycho-pedagogical design, and to adjust the learning setting in response. Although this approach generates data years after the completion of the course, and does not allow to generate immediate feedback for teachers, we argue that the long term impact of higher education courses should not be ignored. Interrogating student perspectives over time can provide crucial information about the impact of an educational psycho-pedagogical design on the students’ future personal and professional learning and lives. Furthermore, considering that the career of a teacher might last several decades, we argue that teachers might be able to use such feedback in a productive way while progressively fine-tuning their professional intervention over time.

Second, from the learners’ point of view, responding to the survey we developed in relation to a course they attended several years before allows them to reflect upon their learning and to become aware of their development. In particular, including information about the transfer of skills acquired during the course represents an opportunity for significant reflection on their long term learning.

Third, in relation to stakeholders such as policy makers, within the Italian national context, higher education assessment tools aimed at specific evaluation of single courses are conspicuously absent. Recent reforms have introduced several assessment tools, but these always target short term effects and the overall evaluation of the whole set of courses comprising the bachelor or master degrees. Tools aimed at assessing long terms effects are still needed. A research approach such as the one proposed in this article triggers specific reflection on the long-term effects of individual courses as well as gleaning insight into the types of courses that most contribute to students’ learning and wellbeing after graduation.

## Data Availability Statement

The datasets generated for this study are available on request to the corresponding author.

## Ethics Statement

Ethical review and approval was not required for the study on human participants in accordance with the local legislation and institutional requirements. The patients/participants provided their written informed consent to participate in this study.

## Author Contributions

ML contributed to the design and conception of the study. ML and RD carried out the data collection and contributed to the analysis and interpretation of the data, and wrote the first draft of the manuscript. GR outlined the theoretical framework. GR, KM, and SA contributed to the interpretation and discussion of the data, and reviewed the manuscript contributing significantly to improve it, in some cases re-writing some sections. All authors contributed to multiple iterations of manuscript revision. Finally, GR took responsibility to finalize the manuscript and act as corresponding author.

## Conflict of Interest

The authors declare that the research was conducted in the absence of any commercial or financial relationships that could be construed as a potential conflict of interest.

## References

[B1] Annese S. Traetta M. (2011). “A methodological approach for blended communities: social network analysis and positioning network analysis,” in *Handbook of Research on Methods and Techniques for Studying Virtual Communities: Paradigms and Phenomena*, ed. Daniel B. K. (Hershey: IGI Global), 103–121. 10.4018/978-1-60960-040-2.ch006

[B2] Annese S. Traetta M. (2012). Distributed participation in blended learning communities: actors, contexts and groups. *Int. J. Web Based Commun.* 8 422–439.

[B3] Annese S. Traetta M. (2018). “A dialogical approach for learning communities between positioning and reformulation,” in *The Dialogical Self Theory in Education: A Multicultural Perspective*, eds Meijers F. Hermans H. (Cham: Springer), 189–209. 10.1007/978-3-319-62861-5_13

[B4] Annese S. Traetta M. Spadaro P. F. (2010). “Blended learning communities: relational and identity networks,” in *Interpersonal Relations and Social Patterns in Communication Technologies: Discourse Norms, Language Structures and Cultural Variables*, eds Park J. Abels E. G. (Hershey: IGI Global), 256–276. 10.4018/978-1-61520-827-2.ch014

[B5] Aronson E. Patnoe S. (1997). *The Jigsaw Classroom: Building Cooperation in the Classroom*, 2nd Edn New York, NY: Addison Wesley Longman.

[B6] Aronson E. Stephan C. Sikes J. Blaney N. Snapp M. (1978). *The Jigsaw Classroom.* Beverly Hills, CA: Sage Pubblications, Inc.

[B7] Astleitner H. (2018). Multidimensional engagement in learning–an integrated instructional design approach. *J. Instr. Res.* 7 6–32.

[B8] Berger P. Luckman T. (1966). *The Social Construction of Reality: A Treatise it’s the Sociology of Knowledge.* New York, NY: Anchor Books.

[B9] Brown A. L. Campione J. C. (1990). “Communities of learning or a content by any other name,” in *Contribution to Human Development*, ed. Kuhn D. (New York, NY: Oxford University Press), 108–126. 10.1159/000418984

[B10] Brown G. T. Harris L. R. (2014). The future of self-assessment in classroom practice: reframing self-assessment as a core competency. *Frontline Learn. Res.* 2 22–30. 10.14786/flr.v2i1.24

[B11] Bruner J. (1996). *The Culture of Education.* Cambridge, MA: Harvard University Press.

[B12] Carless D. (2016). “Scaling up assessment for learning: progress and prospects,” in *Scaling up Assessment for Learning in Higher Education*, eds Carless D. Bridges S. Chan C. Glofcheski R. (Singapore: Springer), 3–17. 10.1007/978-981-10-3045-1_1

[B13] Cook-Sather A. (2002). Authorizing students’ perspectives: toward trust, dialogue, and change in education. *Educ. Res.* 31 3–14. 10.3102/0013189x031004003

[B14] Cucchiara S. Ligorio M. B. Fujita N. (2014). “Understanding online discourse strategies for knowledge building through social network analysis,” in *Innovative Methods and Technologies for Electronic Discourse Analysis*, eds Lim H. Sudweeks F. (Hershey, PA: Information Science Reference), 42–62. 10.4018/978-1-4666-4426-7.ch003

[B15] Dillenbourg P. (1999). *What do you Mean by Collaborative Learning? Cognitive and Computational Approaches.* Oxford: Elsevier, 1–19.

[B16] Engeström Y. (1987). *Learning by Expanding.* Helsinki: OrientaKonsultit.

[B17] Engle R. A. (2006). Framing interactions to foster generative learning: a situative explanation of transfer in a community of learners classroom. *J. Learn. Sci.* 15 451–498. 10.1207/s15327809jls1504_2

[B18] Eom S. B. Ashill N. Wen J. (2006). The determinants of students’ perceived learning outcomes and satisfaction in University online education: an empirical investigation. *Decis. Sci. J. Innov. Educ.* 4 215–235. 10.1111/j.1540-4609.2006.00114.x

[B19] Etzkowitz H. (2008). *The Triple Helix: University-Industry-Government Innovation in Action.* Abingdon: Routledge.

[B20] European Association for Quality Assurance in Higher Education [ENQA] (2005). *Standards and Guidelines for Quality Assurance in the European Higher Education Area.* Available online at: https://media.ehea.info/file/ENQA/05/3/ENQA-Bergen-Report_579053.pdf (accessed March 25, 2018).

[B21] European Association for Quality Assurance in Higher Education [ENQA] (2015). *Standards and Guidelines for Quality Assurance in the European Higher Education Area.* Available online at: http://www.enqa.eu/wp-content/uploads/2015/11/ESG_2015.pdf (accessed March 25, 2018).

[B22] European Commission (2013). *Report to the European Commission on Improving the Quality of Teaching and Learning in Europe’s Higher Education Institutions.* Luxembourg: Publications Office of the European Union.

[B23] Gatignon H. Tushman M. L. Smith W. Anderson P. (2002). A structural approach to assessing innovation: construct development of innovation locus, type, and characteristics. *Manag. Sci.* 48 1103–1122. 10.1287/mnsc.48.9.1103.174

[B24] Gergen K. J. (1999). *An Invitation to Social Construction.* London: Sage.

[B25] Gergen K. J. (2001). Psychological science in a postmodern context. *Am. Psychol.* 56 803–813. 10.1037/0003-066x.56.10.803 11675987

[B26] Gillies R. M. (2007). *Cooperative Learning: Integrating Theory and Practice.* Thousand Oaks, CA: SAGE Publications.

[B27] Glaser B. G. Strauss A. (1967). *The Discovery of Grounded Theory: Strategies of Qualitative Research.* Chicago: Aldine.

[B28] Graham C. R. (2006). “Blended learning systems,” in *Handbook of Blended Learning: Global Perspectives, Local Designs*, eds Bonk C. J. Graham C. R. (San Francisco, CA: Pfeiffer Publishing), 3–21.

[B29] Green L. W. (1977). Evaluation and measurement. some dilemmas for health education. *Am. J. Public Health* 67 155–161. 10.2105/ajph.67.2.155 402085 PMC1653552

[B30] Gursul F. Tozmaz G. B. (2010). Which one is smarter? Teacher or board. *Proc. Soc. Behav. Sci.* 2 5731–5737. 10.1016/j.sbspro.2010.03.936

[B31] Hakkarainen K. (2009). A knowledge-practice perspective on technology-mediated learning. *Int. J. Comput. Support. Collab. Learn.* 4 213–231. 10.1007/s11412-009-9064-x

[B32] Hakkarainen K. Sintonen M. (2002). Interrogative model of inquiry and computer-supported collaborative learning. *Sci. Educ.* 11 25–43.

[B33] Hare A. P. (1994). Types of roles in small groups: a bit of history and a current perspective. *Small Group Res.* 25 443–448.

[B34] Henderson M. Selwyn N. Finger G. Aston R. (2015). Students’ everyday engagement with digital technology in university: exploring patterns of use and ‘usefulness’. *J. High. Educ. Policy Manag.* 37 308–319. 10.1080/1360080x.2015.1034424

[B35] Hutchins E. (2001). “Distributed cognition,” in *International Encyclopedia of the Social & Behavioral Sciences*, eds Smelser N. J. Baltes P. B. (Amsterdam: Elsevier), 2068–2072.

[B36] Kelly A. Lesh R. Baek J. (eds) (2014). *Handbook of Design Research Methods in Education.* New York, ny: Routledge.

[B37] Kelly G. A. (1955). *The Psychology of Personal Constructs.* New York, NY: Norton.

[B38] Kirkpatrick D. L. (1998). *Evaluation Training Programs: The Four Level.* San Francisco, CA: Berrett-Kohler.

[B39] Kivunja C. (2015). Teaching students to learn and to work well with 21st century skills: unpacking the career and life skills domain of the new learning paradigm. *Int. J. High. Educ.* 4 1–11.

[B40] Lai M. Lam K. M. Lim C. P. (2016). Design principles for the blend in blended learning: a collective case study. *Teach. High. Educ.* 21 716–729. 10.1080/13562517.2016.1183611

[B41] Lave J. (1988). *Cognition in Practice: Mind, Mathematics and Culture in Everyday Life.* New York, NY: Cambridge University Press.

[B42] Ligorio M. B. Annese S. (2010). “Blended activity design approach: a method to innovate e-learning for higher education,” in *Psychology Research*, eds Blachnio A. Przepiorka A. Rowiński T. (Warsaw: CSWU Press), 165–188.

[B43] Ligorio M. B. Cucchiara S. (2011). Blended collaborative constructive participation (BCCP): a model for teaching in higher education. *eLearning Papers Presented at 27th Transforming Education Through Technology*, Spain.

[B44] Ligorio M. B. Sansone N. (2009). “Structure of a Blended University course: applying constructivist principles to a blended course,” in *Information Technology and Constructivism in Higher Education: Progressive Learning Frameworks*, ed. Payne C. R. (London: IGI Global), 216–230. 10.4018/978-1-60566-654-9.ch014

[B45] Lucena R. Gitirana V. Trouche L. (2018). “Meta-orchestration instrumental for teacher education,” in *Proceedings of the Re (s) sources 2018 International Conference*, Lyon, 300–303.

[B46] Maul A. Penuel W. R. Dadey N. Gallagher L. P. Podkul T. Price E. (2017). Measuring experiences of interest-related pursuits in connected learning. *Educ. Technol. Res. Dev.* 65 1–28. 10.1007/s11423-016-9453-6

[B47] McGraw-Hill Education (2016). *Workforce Readiness Survey.* New York, NY: McGraw-Hill.

[B48] Mowery D. C. Nelson R. R. Sampat B. N. Ziedonis A. A. (2015). *Ivory Tower and Industrial Innovation: University-Industry Technology Transfer Before and After the Bayh-Dole Act.* Palo Alto, CA: Stanford University Press.

[B49] Mudrack P. E. Farrell G. M. (1995). An examination of functional role behavior and its consequences for individuals in group settings. *Small Group Res.* 26 542–571. 10.1177/1046496495264005

[B50] Muukkonen H. Lakkala M. Hakkarainen K. (2005). Technology-mediation and tutoring: how do they shape progressive inquiry discourse? *J. Learn. Sci.* 14 527–565. 10.1207/s15327809jls1404_3

[B51] Owen J. M. Rogers P. J. (1999). *Program Evaluation : Forms and Approaches.* St. Leonards: Allen & Unwin.

[B52] Potter J. (1996). “Representing reality. discourse, rhetoric and social construction,” in *Handbook of Qualitative Research Methods for Psychology and Social Sciences*, ed. Richardson J. E. (Leicester: British Psychological Society), 136–178.

[B53] Ritella G. Hakkarainen K. (2012). Instrumental genesis in technology-mediated learning: from double stimulation to expansive knowledge practices. *Int. J. Comput. Support. Collab. Learn.* 7 239–258. 10.1007/s11412-012-9144-1

[B54] Ritella G. Ligorio M. B. (2016). Investigating chronotopes to advance a dialogical theory of collaborative sensemaking. *Cult. Psychol.* 22 216–231. 10.1177/1354067x15621475

[B55] Ritella G. Ligorio M. B. Hakkarainen K. (2017). Interconnections between the discursive framing of space-time and the interpretation of a collaborative task. *Learn. Cult. Soc. Interact.* 20 45–57. 10.1016/j.lcsi.2017.08.001

[B56] Ritella G. Sansone N. (2020). Transforming the space-time of learning through interactive whiteboards: the case of a knowledge creation collaborative task. *Qwerty* 15 10.30557/QW000022

[B57] Salanova M. P. (2004). Engagement and burnout: analysing their associated patterns. *Psychol. Rep.* 94 1048–1050. 10.2466/pr0.94.3.1048-1050 15217069

[B58] Säljö R. (2016). “Apps and learning,” in *Apps, Technology and Younger Learners: International Evidence for Teaching*, eds Kucirkova N. Falloon G. (Milton Park: Taylor & Francis).

[B59] Samuel R. Bergman M. M. Hupka-Brunner S. (2013). The interplay between educational achievement, occupational success, and well-being. *Soc. Indic. Res.* 111 75–96. 10.1586/ern.11.50 21539485

[B60] Sansone N. Ligorio M. B. Buglass S. (2016). Peer e-tutoring: effects on students’ participation and interaction style in online course. *Innov. Educ. Teach. Int.* 55 13–22. 10.1080/14703297.2016.1190296

[B61] Scardamalia M. Bereiter C. (2006). “Knowledge building: theory pedagogy, and technology,” in *Cambridge Handbook of the Learning Sciences*, ed. Sawyer K. (New York, NY: Macmillan), 97–118.

[B62] Schleicher A. (2011). *Building a High-Quality Teaching Profession: Lessons from Around the World.* Paris: Organization for Economic Cooperation and Development (OECD).

[B63] Schneider M. Preckel F. (2017). Variables associated with achievement in higher education: a systematic review of meta-analyses. *Psychol. Bull.* 143 565–600. 10.1037/bul0000098 28333495

[B64] Schomaker J. Meeter M. (2015). Short-and long-lasting consequences of novelty, deviance and surprise on brain and cognition. *Neurosci. Biobehav. Rev.* 55 268–279. 10.1016/j.neubiorev.2015.05.002 25976634

[B65] Shotter J. (1993). *Cultural Politics of Everyday Life: Social Constructionism, Rhetoric, and Knowing of the third Kind.* Milton Keyne: Open University Press.

[B66] Smith K. A. Sheppard S. D. Johnson D. W. Johnson R. T. (2005). Pedagogies of engagement: classroom-based practices. *J. Eng. Educ.* 94 87–101. 10.1002/j.2168-9830.2005.tb00831.x

[B67] Steeples C. Goodyear P. Mellar H. (1994). *‘Flexible Learning in Higher Education: The use of Computer-Mediated Communications’, Computers and Education.* Oxford: Pergamon Press.

[B68] Strijbos J. W. Weinberger A. (2010). Emerging and scripted roles in computer-supported collaborative learning. *Comput. Hum. Behav.* 26 491–494. 10.1016/j.chb.2009.08.006

[B69] Suchman L. (1987). *Plans and Situaded Actions.* Cambridge: Cambridge University Press.

[B70] Tadesse T. Gillies R. M. (2015). Nurturing cooperative learning pedagogies in higher education classrooms: evidence of instructional reform and potential challenges. *Curr. Issues Educ.* 18 1–18.

[B71] Topping K. (2005). Trends in peer learning. *Educ. Psychol.* 25 631–645. 10.1080/01443410500345172

[B72] Topping K. J. (2003). “Self and peer assessment in school and university: reliability, validity and utility,” in *Optimizing New Modes of Assessment: In Search of Qualities and Standards*, eds Segers M. Dochy F. Cascallar E. (Dordrecht: Kluwer Academic), 55–87. 10.1007/0-306-48125-1_4

[B73] van Dinther M. Dochy F. Segers M. (2015). The contribution of assessment experiences to student teachers’ self-efficacy in competence-based education. *Teach. Teach. Educ.* 49 45–55. 10.1016/j.tate.2015.02.013

[B74] Vygotsky L. S. (1986). *Thought and Language.* Cambridge, MA: MIT Press.

[B75] Wanner T. Palmer E. (2015). Personalising learning: exploring student and teacher perceptions about flexible learning and assessment in a flipped university course. *Comput. Educ.* 88 354–369. 10.1016/j.compedu.2015.07.008

[B76] Wenger E. C. (1998). *Communities of Practice. Learning, Meaning, and Identity.* New York, NY: Cambridge University Press.

[B77] Zhao Y. (2015). A world at risk: an imperative for a paradigm shift to cultivate 21st century learners. *Society* 52 129–135. 10.1007/s12115-015-9872-8

[B78] Zigurs I. Kozar K. A. (1994). An exploratory study of roles in computer-supported groups. *MISQuarterly* 18 277–297.

[B79] Zimmerman B. J. (2001). “Theories of self-regulated learning and academic achievement: an overview and analysis,” in *Self-Regulated Learning and Academic Achievement: Theoretical Perspectives*, eds Zimmerman B. J. Schunk D. H. (Mahwah, NJ: Lawrence Erlbaum Associates Publishers), 1–37.

