# Supplementary material for: Remembering, Reflecting, Reframing: Examining Students’ Long-Term Perceptions of an Innovative Model for University Teaching
Source: Front Psychol. 2020 Mar 31;11:565. doi: 10.3389/fpsyg.2020.00565 (PMC7137826; doi:10.3389/fpsyg.2020.00565)
Supplement: Supplementary file 1 [file Data_Sheet_1.pdf]

1 Appendix 1: The Questionnaire

Welcome

We are conducting a study concerning the course "Educational Psychology and E-learning" of the University of Studies of Bari. We ask you 15 minutes of your time to answer the questions in the most spontaneous way possible, considering that there are no right or wrong answers and that the questionnaire is anonymous.

We appreciate your cooperation.

1) Age: ___________________
2) Genere: Male Female

3) What do you remember from the course?

____
____
____
____
____

4) Evaluate using a scale from 1 to 3 (1 = a little, 3 = a lot) how much you think you have reused the following skills learnt during the course

4 a. How to organize my work in view of a common goal

- A little
- Enough
- A lot

4 b. How to organize group work in view of a common goal

- A little
- Enough
- A lot

4 c. How to communicate effectively for the creation of collaborative products

- A little
- Enough
- A lot

4 d. How to communicate effectively within a working group

- A little
- Enough
- A lot

4 e. How to negotiate between different points of view

- A little
- Enough
- A lot

4 f. How to observe group dynamics

- A little
- Enough

- A lot

4 g. How to support group dynamics
- A little
- Enough
- A lot

4 h. How to use technologies for learning and training purposes
- A little
- Enough
- A lot

4 i. How to create collaborative products (maps, plans, reports etc.)
- A little
- Enough
- A lot

4 j. Be able to summarize and re-elaborate topics
- A little
- Enough
- A lot

4 k. How to write academic texts
- A little
- Enough
- A lot

4 l. How to find useful and reliable information on the web
- A little
- Enough
- A lot

4 m. How to manage work deadlines
- A little
- Enough
- A lot

4 n. How to compare similar products prepared by different people / groups
- A little
- Enough
- A lot

4 o. How to evaluate online courses
- A little
- Enough
- A lot

4 p. How to design online courses
- A little
- Enough
- A lot

4 q. Know how to operationalize theoretical constructs related to e-learning
- A little
- Enough
- A lot

4 r. Team working capacity
- A little

- Enough
- A lot

4 s. How to cover different roles in collaborative groups
- A little
- Enough
- A lot

4 t. Capacity to reflect on group dynamics
- A little
- Enough
- A lot

4 u. Capacity to reflect on your own learning processes
- A little
- Enough
- A lot

4 v. How to enhance my own skills
- A little
- Enough
- A lot

4 w. How to self-evaluate
- A little
- Enough
- A lot

4 x. Be able to meet deadlines
- A little
- Enough
- A lot

4 y. How to deal with unexpected and sudden changes during group work
- A little
- Enough
- A lot

4 z. Be flexible in facing problematic situations that may arise during group work
- A little
- Enough
- A lot

4 aa. Other. To specify
____
____
____
____

5) In which context did you use them?

5.a) online contexts
- other university courses
- other types of courses
- work contexts
- other

5.b) offline contexts
- other university courses
- other types of courses
- work contexts

- other

6) In what year did you attend the course “Educational Psychology and E-learning"
