# Supplementary material for: Remembering, Reflecting, Reframing: Examining Students’ Long-Term Perceptions of an Innovative Model for University Teaching
Source: Front Psychol. 2020 Mar 31;11:565. doi: 10.3389/fpsyg.2020.00565 (PMC7137826; doi:10.3389/fpsyg.2020.00565)
Supplement: Supplementary file 2 [file Data_Sheet_2.pdf]

Appendix 2: Summary of data

Table 1: Categorization of memories

	didactic methods	technological tools	competences	group dynamics	learning contents	general criticisms	TOT
2005-2008	5	1	1	1	1	3	12
	42%	8%	8%	8%	8%	25%	
2009-2011	8	4	1	1	3	11	28
	29%	14%	4%	4%	11%	39%	
2012-2015	24	3	5	6	14	13	65
	37%	5%	8%	9%	22%	20%	
TOT	37	8	7	8	18	27	105
	35%	8%	7%	8%	17%	26%	

Table 2: Percentage of students reporting to have reused each type of skills and competences

	2005-2008			2009-2011			2012-2015		
	a little	enough	a lot	a little	enough	a lot	a little	enough	a lot
Organizational	1%	25%	74%	7%	31%	62%	2%	25%	73%
Communication	0%	12%	88%	6%	37%	57%	0%	22%	78%
Academic	13%	18%	69%	20%	31%	49%	7%	25%	68%
Group dynamics	10%	32%	58%	9%	33%	58%	4%	25%	71%
E-learning	23%	19%	58%	46%	19%	35%	20%	30%	50%
Self-assessment	0%	12%	88%	19%	29%	52%	2%	24%	74%

Table 3: Contexts where skills and competences have been reused

	Other university courses	Other type of training	Work contexts	Other	TOT
online	26	33	30	7	96
	27%	34%	31%	7%	
offline	27	20	44	5	96
	28%	21%	46%	5%	
